# *Hongiastoma zhangbuensis*, a New Species of the Subfamily Acrossocheilinae (Teleostei: Cyprinidae) from South China †

**DOI:** 10.3390/ani15172563

**Published:** 2025-08-31

**Authors:** Lan-Ping Zheng, Wei-Tao Chen

**Affiliations:** 1 College of Traditional Chinese Medicine, Yunnan University of Chinese Medicine, Kunming 650500, China; 2 Pearl River Fisheries Research Institute, Chinese Academy of Fishery Sciences, Guangzhou 510380, China

**Keywords:** morphology, molecular phylogenetics, COI, Cypriniformes

## Abstract

*Hongiastoma zhangbuensis* sp. nov. is described from a tributary of the upper reach of the Pearl River basin based on the morphological and molecular data. It is distinguished from all known species within the subfamily Acrossocheilinae by having a combination of the following characteristics: a crescent horny sheath on the lower jaw, a less prominent lower lip limited to the corners of the mouth, a serrated posterior edge of the last simple dorsal-ray, 47–50 lateral-line scales, 9 scales above the lateral line, 16–18 circumpeduncular scales, and 16–18 pre-dorsal midline scales. The molecular phylogeny revealed that *H. zhangbuensis* sp. nov. was a sister taxon to *Hongiastomata argentatum*, with a genetic distance ranging from 10.49% to 10.68%. Together, they formed a lineage at the base of the Acrossocheilinae clade. Furthermore, the interspecific genetic distances between the new species and those of *Onychostoma* sensu lato ranged from 9.9 to 13.4%, indicating significant genetic divergence between this new species and all known species within *Onychostoma* sensu lato. Therefore, both morphological and molecular data support the recognition of this species as new.

## Simple Summary

The family Cyprinidae has significant economic value. The genus *Hongiastoma* belongs to the subfamily Acrossocheilinae within the family Cyprinidae, which was recently established and is currently monotypic. A new species, *Hongiastoma zhangbuensis* sp. nov. (urn:lsid:zoobank.org:act:4F2A8E7C-4DD6-4413-81FB-50B833E97135) is described and illustrated from the Pearl River basin in this study. This is the first record of the genus *Hongiastoma* in China.

## 1. Introduction

Cyprinidae is the largest family within Cypriniformes, and it contains more than 1700 valid species [1]. At present, Cyprinidae is divided into 10 subfamilies: Acrossocheilinae, Barbinae, Cyprininae, Labeoninae, Probarbinae, Torinae, Smiliogastrinae, Spinibarbinae, Schizothoracinae, and Schizopygopsinae [1]. The genus *Hongiastoma* (Hoang and Nguyen, 2025) belongs to the subfamily Acrossocheilinae within the family Cyprinidae [2]. It was recently established, with *Varicorhinus argentatus* (Nguyen and Doan, 1969) designated as its type species [2]. This species has long been regarded as a member of *Onychostoma* (Günther, 1896) [3] and was once considered to be a possible synonym of *Onychostoma lepturus* (Boulenger, 1900) [4,5]. However, Roberts and Catania [6] designated the lectotypes for this species and determined its species status. The genus *Onychostoma* sensu lato has been recorded to have more than 20 species, and the overwhelming majority species are distributed in China [7,8]. However, several studies of molecular phylogeny in the past have indicated that *Onychostoma* was not monophyly, indicating the necessity for taxonomic revision of this genus [9,10]. Recently, Hoang et al. [2] conducted a taxonomic revision of the genus *Onychostoma* and established four additional genera for the species previously classified under *Onychostoma*: *Angustistoma*, *Hongiastoma*, *Scaphostoma*, and *Scaphesthes.* These five genera align with five different lineages in the molecular phylogeny. *Hongiastoma* shares the horny sheath on lower jaw and eight branched dorsal-fin rays with *Onychostoma*, *Angustistoma*, *Scaphostoma*, and *Scaphesthes*. However, it is located in the basal lineage of Acrossocheilinae in molecular phylogeny and can be distinguished from the species of these four genera by a series of morphological characteristics: a crescent horny sheath on the lower jaw (vs. horseshoe-shaped or ends that bend backward in *Angustistoma*, *Onychostoma,* and *Scaphesthes*); more than 7 scales above the lateral line (vs. 5–6 in *Scaphesthes*); and the absence of a longitudinal black stripe along the lateral body (vs. presence in *Scaphostoma*) [2]. So far, only one species has been recorded in the genus *Hongiastoma*, and it is only known to be distributed in the Red River basin of Hoa Binh Province, Vietnam currently.

Recently, a batch of specimens was collected from the Pearl River basin in Guizhou Province, China, exhibiting a horny sheath on the lower jaw, eight branched dorsal-fin rays, and other typical characteristics of *Onychostoma* sensu lato. However, these specimens are morphologically distinct from known species within *Onychostoma* sensu lato, indicating they represent an undescribed new species. To confirm its species status and validate this classification, we integrated molecular data for analysis in this study. The molecular results also support that this species is an independent new lineage. Therefore, we present a description of this new species, along with the morphological comparison and molecular analysis within the subfamily Acrossocheilinae.

## 2. Materials and Methods

### 2.1. Sampling

Samples were collected during fish surveys in the Pearl River basin in 2024. The fish were first anesthetized with clove oil, then preserved in 10% formalin, and subsequently transferred to 75% ethyl alcohol. The specimens are deposited at the Kunming Institute of Zoology, Chinese Academy of Sciences, in Kunming (KIZ).

### 2.2. Morphological Examination

Counts and measurements mainly adhere to Kottelat [11], with counts of fin ray and lateral scales following Chu and Chen [12]. The measurements of pre-dorsal, pre-pectoral, pre-pelvic, and pre-anal lengths, were conducted according to Zheng et al. [13]. Measurements were taken using digital calipers with an accuracy of 0.1 mm, employing a point-to-point method. Pharyngeal dentition and vertebrae were scanned using a Hiscan XM Micro-CT System (Haesfeld, Suzhou, China) with a 4 μm pixel resolution, and the cross-sections of the specimen were reconstructed using the associated software. Three-dimensional renderings were created, visualized, and manipulated using Hiscan viewer. The examined specimens are kept in the Kunming Institute of Zoology, Chinese Academy of Sciences, in Kunming (KIZ). Abbreviations used in the text are as follows: TL for total length, SL for standard length, and HL for lateral head length.

### 2.3. Molecular Analysis

The genomic DNA was extracted from fin clips preserved in 95% ethanol. The gene of cytochrome oxidase subunit I (COI) was used in this study. The details of the primer information for PCR amplification of the COI gene were provided in Zheng et al. [14]. A total of 90 sequences of COI were downloaded from GenBank. The species of *Labeo* were used as the root outgroup according to Yang et al. [9]. Sequencing was performed directly using the corresponding PCR primers. PCR products were purified via spin columns. Purified PCR products were sequenced in both forward and reverse directions using the sequencing services of BigDye Terminator v3.1 on ABI PRISM 3730 (Applied Biosystems, Carlsbad, CA, USA) following the manufacturer’s instructions. All sequence accession numbers are given in Table S1.

Sequences were aligned using MAFFT v7.475 [15], then trimmed using trimAl v1.4 [16]. The pairwise genetic distances were calculated using MEGA X based on the p-distance model [17]. Phylogenetic reconstruction was carried out with maximum likelihood (ML) approaches. ML analysis was performed using IQ-TREE v2.1.4 [18] based on the best-substitution model selected by ModelFinder in the IQ-TREE package [19]. The BI analysis was performed using MrBayes v3.2.7 [20], with the best-fit nucleotide substitution model also determined using ModelFinder v1.6.12. Four chains (three hot and one cold) were run for 5,000,000 generations, sampling trees every 100 generations and with the first 12,500 generations discarded as burn-in. Convergence was confirmed by ascertaining that the average standard deviation of split frequencies was below 0.01. The phylogenetic trees were viewed and edited using FigTree v1.4.4 [21].

## 3. Results

### 3.1. Taxonomy

*Hongiastoma* (Hoang and Nguyen 2025)

Diagnosis. *Hongiastoma* can be distinguished from other genera within *Onychostoma sensu lato* by the following unique combination of characteristics: a crescent horny sheath on the lower jaw (vs. horseshoe-shaped or ends that bend backward in *Angustistoma, Onychostoma*, and *Scaphesthes*); more than 7 scales above the lateral line (vs. 5–6 in *Scaphesthes*); and the absence of a longitudinal black stripe along the lateral body (vs. presence in *Scaphostoma*).

**Figure 1:**
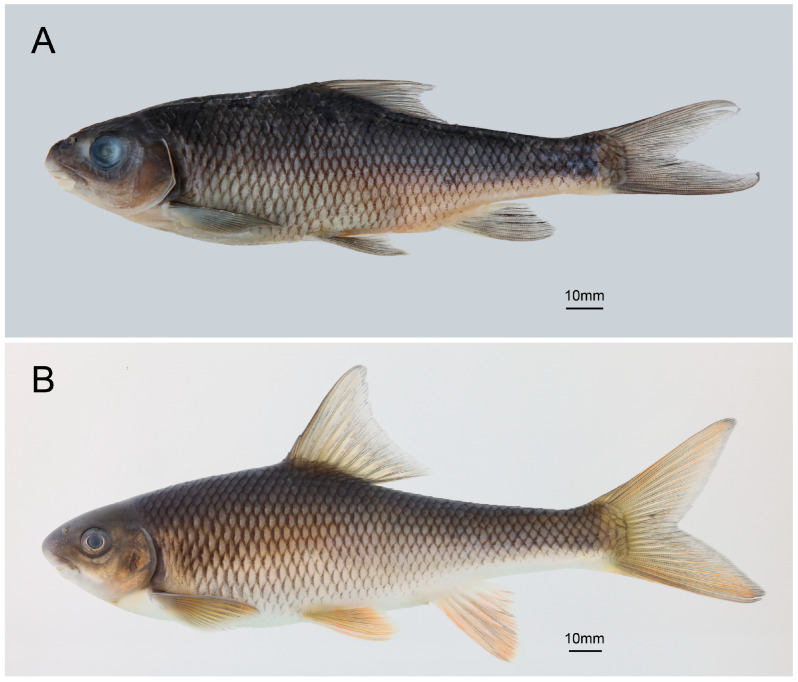
Lateral view of *Hongiastoma zhangbuensis* sp. nov. (**A**) KIZ 2024011623, paratype, 144.6 mm SL; (**B**) not preserved, 217.2 mm TL.

*Hongiastoma zhangbuensis* sp. nov. (Figure 1)

Holotype. KIZ 2024011621, 189.9 mm SL; China: Guizhou: Pingtang County: Zhangbu River, a tributary of Pearl River at Zhangbu town, 26°3′38″ N 107°3′16″ E; L.-P. Zheng, 3 October 2024.

Paratypes. KIZ 2024011622-1625, 4, 110.6-144.9 mm SL; collected with the holotype.

Diagnosis. *Hongiastoma zhangbuensis* can be distinguished from *Hongiastoma argentatum* by the following unique combination of characteristics: last simple dorsal-ray hard and serrated posteriorly (vs. slender and smooth), dorsal-fin origin positioned anterior to (vs. behind/opposite to) pelvic-fin insertion, and more (9 vs. 7) scales above the lateral-line.

**Figure 2:**
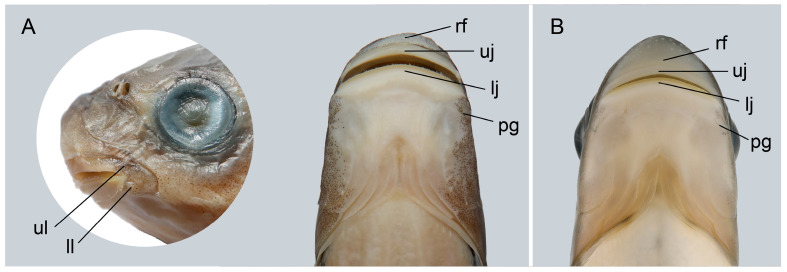
Ventral view of mouth of *Hongiastoma zhangbuensis* sp. nov. (**A**) KIZ 2024011623, paratype, 144.6 mm SL; (**B**) not preserved, 217.2 mm TL. Abbreviations: ul, upper lip; ll, lower lip; rf, rostral fold; uj, upper jaw; lj, lower jaw; pg, post-labial groove.

Description. Morphometric data are listed in Table 1. Body elongated, laterally compressed and moderately deep. Deepest part of body usually in front of dorsal-fin origin, and the least depth slightly anterior to the caudal-fin base (Figure 1A,B). Head rounded and depth greater than its width. Snout blunt and rounded when laterally viewed, with its length approximately equal to the post-orbital length. Eyes large, in the anterior half of the head, close to the dorsal profile. Interorbital space slightly convex. Mouth inferior and transverse. Mouth wide, its width equal to the width of the head at that point. Rostral fold simple and downwards, covering the base of the upper lip. Upper lip thin, smooth, entirely enclosing upper jaw, laterally confluent with lower lip around corners of mouth. Lower lip simple, restricted only to the side of lower jaw, adnate with jaw and not free anteriorly. Lower jaw bearing sharp horny sheath on the cutting edge. Post-labial grooves short, restricted in the corner of mouth, its width shorter than the eye diameter. No barbels (Figure 2A,B).

**Figure 3:**
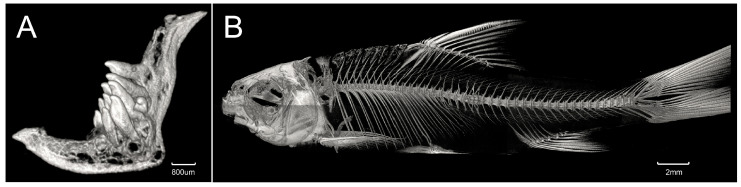
Pharyngeal dentition (**A**) and vertebrae (**B**) of the CT scan of *Hongiastoma zhangbuensis* sp. nov. paratype, KIZ 2024011625, 110.6 mm SL.

Dorsal fin with 4 simple and 8 branched rays; origin slightly anterior to the pelvic-fin insertion, closer to the snout tip than to the caudal-fin base; distal edge slightly concave; last simple dorsal-ray hard and serrated posteriorly. Pectoral fin with 1 simple and 12 (2) or 13 (3) branched rays; second branched ray longest, tip of adpressed fin extending halfway to the pelvic-fin insertion. Pelvic fin with 1 simple and 7 (1) or 8 (4) branched rays, inserted posterior to vertical through the dorsal-fin origin; first and second branched ray longest, extending beyond halfway to the anal-fin origin when depressed. Anal fin with 3 simple and 5 (5) branched rays, origin closer to the pelvic-fin insertion than to the caudal-fin base; distal margin slightly concave. Anus close to the anal-fin origin, not exceeding the width of one scale. Caudal fin with 9 + 8 branched rays, deeply forked, longest rays approximately two times as long as the shortest ones; upper lobes shorter than lower lobes in length, both with pointed tips. Body scales moderately sized; belly scaled, and scales on chest anterior to the pectoral fin origin embedded beneath the skin. Lateral line complete, with 47 (1), 49 (2), or 50 (2) scales, slightly sloping downwards anterior to middle of pectoral fin, then nearly horizontal posterior to the pelvic fin. Scale rows between the lateral line and origin of the dorsal fin 9 (5), and 4 1/2 (5) scales between the lateral line and pelvic-fin insertion. Pre-dorsal midline scales 16 (1), 17 (3), or 18 (1), slightly smaller than those on the flank, not embedded beneath the skin. Circumpeduncular scales 16 (2) or 18 (3). Vertebrae: 4 + 41; pharyngeal teeth triserial as 2, 3, 4−4, 3, and 2 (1 specimens under micro-CT examination; Figure 3A,B).

Coloration. In preserved specimens, head and body dark grey dorsally, light yellow ventrally. Each scale on the side of the body has black pigment spots along its edges, which cluster together to form diamond-shaped patterns on the sides. Fin-rays black, fin membranes hyaline. When alive, head and body dark grey dorsally, light yellow ventrally, while pectoral-, ventral-, anal-, and caudal fins are orange (Figure 1B).

**Figure 4:**
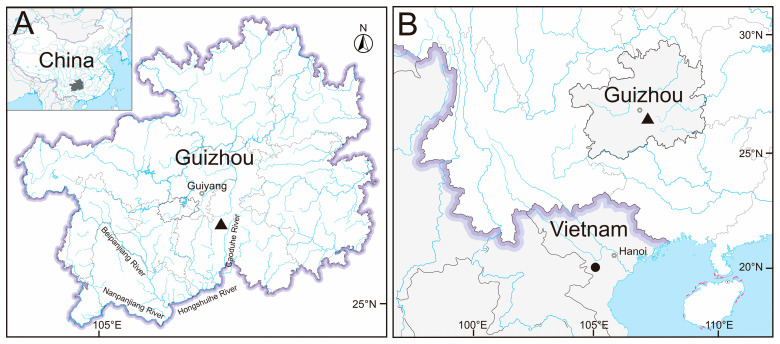
Distribution of *Hongiastoma zhangbuensis* sp. nov. and *Hongiastoma argentatum***.** (**A**) shows the type locality for *H. zhangbuensis* sp. nov.; the gray area in the upper left corner marks the location of Guizhou Province. (**B**) indicates the type locality for two species of *Hongiastoma*. The triangle indicates the collection location of *H. zhangbuensis* sp. nov., while the solid circle represents the type locality of *H. argentatum*.

**Figure 5:**
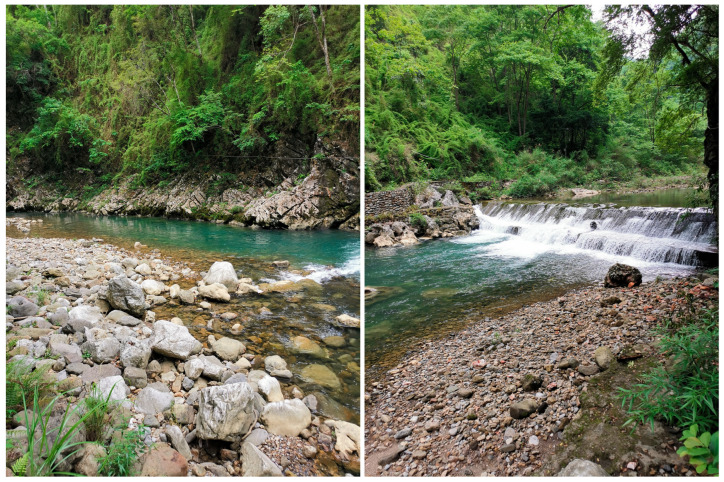
Habit of *Hongiastoma zhangbuensis* sp. nov in the Zhangbu River.

Distribution and Ecology. *Hongiastoma zhangbuensis* sp. nov. is presently known only from the Zhangbu River, a tributary of the Caodu River in Pingtang County, Guizhou Province, China, which flows into the Hongshui River, the upper reach of the Pearl River (Figure 4). This species mainly inhabits in the middle to lower layer of the river, and other species that inhabit the same waters include *Onychostoma yunnanense* (Regan 1904), *Discogobio laticeps* (Chu, Cui, and Zhou, 1993), *Zacco platypus* (Temminck and Schlegel, 1846), *Opsariichthys bidens* (Günther, 1873), and *Hemibarbus medius* (Yue, 1995; Figure 5).

Etymology. Named for the type locality, the Zhangbu River. To be treated as a noun in apposition.

### 3.2. Molecular Results

**Figure 6:**
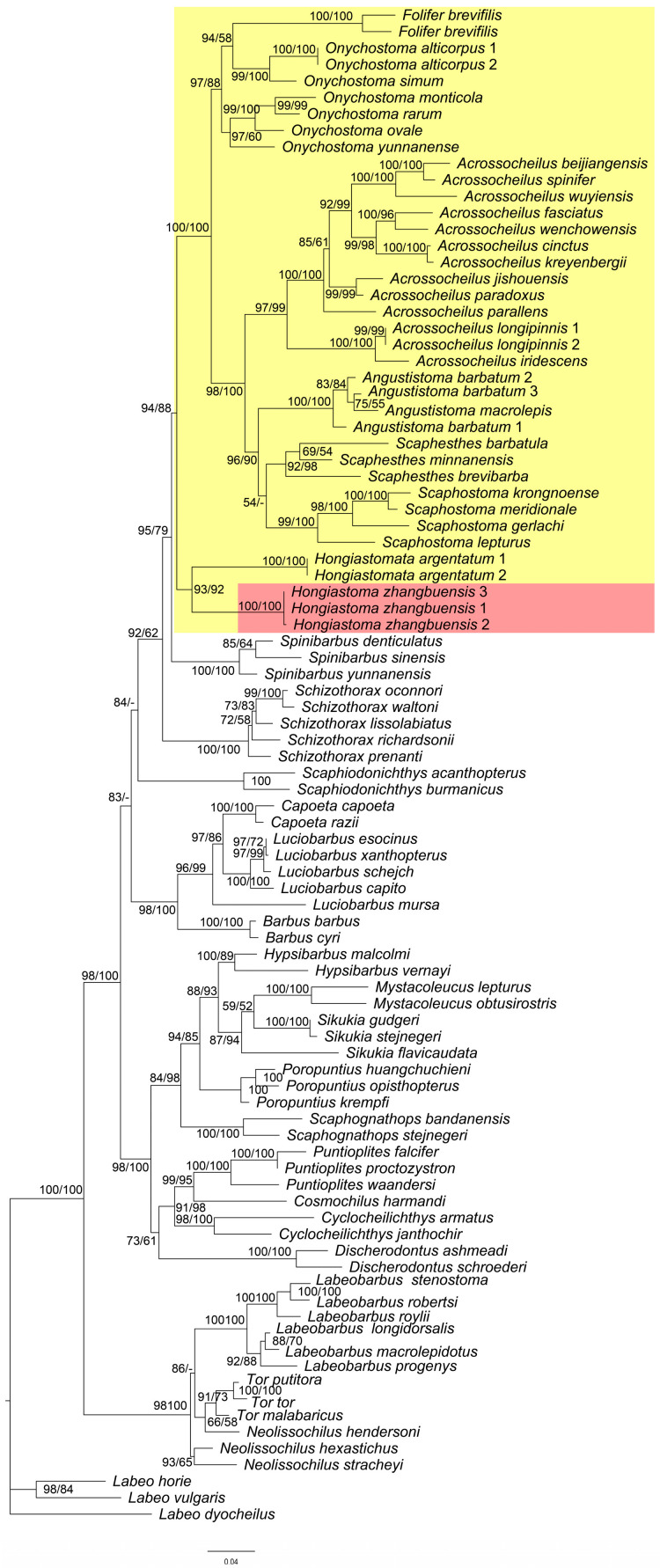
Phylogenetic tree constructed based on COI gene sequences. Nodal numbers are ML bootstrap values and BI posterior probability values, respectively. Only values above 50% are given. Yellow block indicates the subfamily Acrossocheilinae and red indicates the new species.

Sequences of the COI gene from 38 species of Acrossocheilinae and 55 species of other cyprinid were used to construct phylogenetic trees. TVM + F + I + G4 and GTR + F + I + G4 was chosen as the best fit model for the ML and BI analyses, respectively. The phylogenetic analysis demonstrated that the subfamily Acrossocheilinae formed a monophyletic clade. Additionally, the species of *Hongiastoma* were grouped into a separate monophyletic cluster, situated at the basal position within Acrossocheilinae. Additionally, the genera *Angustistoma*, *Scaphostoma*, and *Scaphesthes* were clustered together in one clade, each forming three distinct sub-clades. Furthermore, *Folifer brevilis* was found to be nested within the clade of *Onychostoma*, resulting in the formation of a non-monophyletic clade of *Onychostoma* (Figure 6).

The interspecific genetic distances for *Angustistoma*, *Onychostoma*, *Scaphostoma*, and *Scaphesthes* were 1.8–2.5%, 4.1–10.5%, 5.8–8.9%, and 1.4–8.2%, respectively. The genetic distance between *H. zhangbuensis* sp. nov. and *H. argentatum* were 10.5–10.7%. The values for *H. zhangbuensis* sp. nov. compared to *Angustistoma*, *Onychostoma*, *Scaphostoma*, and *Scaphesthes* ranged from 11.7% to 13.2%, 9.9% to 11.7%, 10.9% to 12.8%, and 12.0% to 13.4%, respectively (Table S2).

## 4. Discussion

According to Yang et al. [8], the subfamily Acrossocheilinae once included three genera: *Acrossocheilus* (Oshima, 1919), *Folifer* (Wu, 1977), and *Onychostoma*. Subsequently, the species of *Onychostoma* were allocated into *Angustistoma*, *Hongiastoma*, *Onychostoma*, *Scaphostoma*, and *Scaphesthes,* respectively [1]. *Acrossocheilus*, *Folifer*, and *Onychostoma* sensu lato can be distinguished from each other by the differences in mouth morphology. *Folifer* has thick, fleshy lips, featuring two lateral lobes and a median lobe on the lower jaw, while *Acrossocheilus* features fleshy lips with a medially disrupted lower lip. In contrast, *Onychostoma* sensu lato possesses simple lips and a horny sheath on the lower jaw [2,7]. Because *Onychostoma* sensu lato share some common characteristics, such as a horny sheath on the lower jaw and eight branched dorsal-fin rays, it was once considered a genus. However, previous research indicated that *Onychostoma* sensu lato comprises at least four clades based on the molecular phylogeny [2,10]. Consequently, *Angustistoma*, *Hongiastoma*, *Scaphostoma*, and *Scaphesthes* were established and separated from *Onychostoma*. *Angustistoma*, *Hongiastoma*, *Onychostoma*, *Scaphostoma*, and *Scaphesthes* share the horny sheath on the lower jaw, but they can be distinguished from each other by the morphological characteristics [2]. The genus of *Hongiastoma* was established by Hoang et al. (2025) based on *Varicorhinus argentatus* (Nguyen and Doan, 1969) [2], and it currently includes only this single recorded species. *Hongiastoma zhangbuensis* sp. nov. can be distinguished from *H. argentatum* by last simple dorsal-ray hard and serrated posteriorly (vs. slender and smooth), and the dorsal-fin origin anterior to (vs. behind/opposite to) pelvic-fin insertion.

Among these genera, *Hongiastoma* is most similar to *Scaphostoma*, because they share the crescent horny sheath on the lower jaw. At present, there are six species within *Scaphostoma*: *Scaphostoma annamense* (Hoang, Pham, and Tran, 2025), *Scaphostoma fusiforme* (Kottelat, 1998), *Scaphostoma gerlachi* (Peters, 1881), *Scaphostoma lepturus* (Boulenger, 1900), *Scaphostoma meridionale* (Kottelat, 1998), and *Scaphostoma krongnoense* (Hoang, Pham, and Tran, 2015). *Hongiastoma zhangbuensis* sp. nov. can be distinguished from all species of *Scaphostoma* by the absence of a longitudinal black stripe along the lateral body (vs. presence), having more scales above the lateral line (9 vs. 6.5–7.5), and further distinguished from *S. lepturus* and *S. meridionale* by the last simple dorsal-ray hard and serrated posteriorly (vs. slender and smooth). Additionally, *H. zhangbuensis* sp. nov. can be further distinguished from *S. annamense* by more pre-dorsal scales (16–18 vs. 12–14).

Currently, a total of nine species of *Onychostoma* sensu lato are distributed in the Pearl River, comprising one species of *Angustistoma*, five species of *Onychostoma*, two species of *Scaphostoma*, and one species of *Scaphesthes*: *Angustistoma barbatum*, *Onychostoma fangi*, *Onychostoma ovale*, *Onychostoma rarum*, *Onychostoma simum*, *Onychostoma yunnanense*, *S. gerlachi*, *S. lepturus*, and *Scaphesthes barbatula*. *Hongiastoma zhangbuensis* sp. nov. can be distinguished from *Angustistoma barbatum* by the absence of black stripes along the lateral body (vs. present), and having more scales above the lateral line (9 vs. 6–7.5). *Hongiastoma zhangbuensis* sp. nov. can be distinguished from *Onychostoma* by having more scales above the lateral line (9 vs. 6 in *O. fangi*; 7–8 in *O. ovale*; 6.5–8 in *O. rarum*; 7–8 in *O. simum*; 6.5–7.5 in *O. yunnanense*). Additionally, it can be further distinguished from *O. ovale* and *O. rarum* by more lateral line scales (47–50 vs. 43–45 in *O. ovale*; 43 in *O. rarum*), and further from *O. fangi* by more circumpeduncular scales (16–18 vs. 12). The distinguished characteristics between *H. zhangbuensis* sp. nov. and the species of *Scaphostoma* have been stated in the above text. *Hongiastoma zhangbuensis* sp. nov. can be distinguished from *S. barbatula* by having more scales above the lateral line (9 vs. 6–6.5), more pre-dorsal scales (16–18 vs. 12–15), and last simple dorsal-ray hard and serrated posteriorly (vs. slender and smooth).

The phylogenetic analysis derived from the COI gene indicated that *H. zhangbuensis* sp. nov. and *H. argentatum* formed a monophyletic linage together, and located at the basal position within Acrossocheilinae. Although these two species share a horny sheath on the lower jaw with *Onychostoma*, *Angustistoma*, *Scaphostoma*, and *Scaphesthes*, they do not form a monophyletic group at the molecular level. It is suggested that the horny sheath on the lower jaw is a trait resulting from convergent evolution. It is no coincidence that the genus *Scaphiodonichthys* within Cyprininae also possess a similar horny sheath on the lower jaw, but it possesses more branched dorsal-fin rays than *Onychostoma* sensu lato (10–14 vs. 8). The genus *Scaphiodonichthys* was initially placed in the subfamily Barbinae but was later revised to the subfamily Cyprinidae based on the molecular results [9]. The above taxonomic revisions also confirm that the horny sheath on lower jaw is a trait that has emerged through convergent evolution. Therefore, when conducting taxonomy, we should not rely solely on a single characteristic, but rather consider it in conjunction with other characteristics comprehensively. Additionally, the molecular analysis revealed that the species of *Onychostoma* and *Folifer brevifilis* formed a lineage together. This finding aligns with the results of Zheng et al. [10]. *Onychostoma* is not monophyletic, indicating that the lineage represented by *Onychostoma rarum* could potentially be a new genus.

From the perspective of genetic distance, the minimum genetic distance between *H. zhangbuensis* sp. nov. and the species of *Onychostoma* is closer to the maximum interspecific genetic distances within the genus *Onychostoma*, which can be attributed to its polyphyly. Apart from *Onychostoma*, the genetic distances between *H. zhangbuensis* sp. nov. and the species of *Angustistoma*, *Scaphostoma*, and *Scaphesthes* are significantly greater than the interspecific genetic distances within those three genera. Furthermore, the genetic distances between *H. zhangbuensis* sp. nov. and *H. argentatum* are also significantly greater than the interspecific genetic distances of the three genera. In other words, the new species exhibits significant genetic divergence from the species of *Onychostoma* sensu lato. Therefore, the molecular analysis supports the classification of this species as a new one.

## 5. Conclusions

A new species of the genus *Hongiastoma*, belonging to the subfamily Acrossocheilinae within the family Cyprinidae, has been illustrated and described herein based on phylogenetic and morphological evidence. This new species is only known to occur in the upper reach of the Pearl River and it is the first discovery and record of the genus *Hongiastoma* in China. The discovery of this new species enhances our understanding of the cyprinid divergence and the species diversity within the Pearl River basin.

## 6. Nomenclatural Acts

This work and the nomenclatural acts have been registered in ZooBank, the online registration system for ICZN. The ZooBank LSIDs (Life Science Identifiers) can be resolved and the associated information viewed through any standard web browser by appending the LSID to the prefix “http://zoobank.org/”. The LSID for this publication is: urn:lsid:zoobank.org:pub:23BA6991-7E7D-4B68-BDA8-880CDBEFF381. The LSID for the new species is: urn:lsid:zoobank.org:act:4F2A8E7C-4DD6-4413-81FB-50B833E97135.

## Figures and Tables

**Table 1:** Morphometric data for *Hongiastoma zhangbuensis* sp. nov.

	Holotype	Range (*n* = 5)	Mean ± SD
In percent of standard length			
Body depth	26.1	26.1–29.0	27.6 ± 1.40
Head length	21.6	21.6–23.2	22.6 ± 0.62
Head depth	19.9	19.7–21.6	20.6 ± 0.87
Head width	15.2	14.2–15.7	14.9 ± 0.54
Dorsal-fin length	31.3	25.2–31.3	27.7 ± 2.46
Pectoral-fin length	21.0	20.2–21.8	21.2 ± 0.66
Pelvic-fin length	19.7	18.5–20.9	20.0 ± 0.95
Anal-fin length	22.5	19.1–22.5	21.3 ± 1.28
Caudal peduncle length	21.5	20.1–21.8	20.9 ± 0.72
Caudal peduncle depth	8.9	8.9–10.5	10.1 ± 0.67
Predorsal length	44.7	44.7–49.3	47.0 ± 1.70
Prepectoral length	21.4	21.4–24.3	22.7 ± 1.20
Prepelvic length	48.6	48.1–49.6	48.7 ± 0.59
Preanal length	70.1	70.1–72.7	72.0 ± 1.06
In percent of lateral head length			
Snout length	38.7	35.5–42.4	39.8 ± 2.64
Head depth	92.2	86.1–94.1	91.4 ± 3.06
Eye diameter	24.7	24.7–28.4	26.3 ± 1.50
Interorbital width	51.7	47.4–51.7	49.6 ± 1.64

## Data Availability

The sequences obtained in this study were deposited in GenBank under the accession number PV789635–PV789637. The downloaded sequences used for tree construction and photos are presented in the text. The morphometric and meristic raw data presented in this study are available upon request from the corresponding author.

## Supplementary Materials

The following supporting information can be downloaded at: https://www.mdpi.com/article/10.3390/ani15172563/s1, Table S1: Species list for phylogenetic analysis in this study; Table S2: Genetic distance of COI gene among the species of *Angustistoma*, *Hongiastoma*, *Onychostoma*, *Scaphostoma*, and *Scaphesthes* within Acrossocheilinae.
