# Supplementary material for: Hongiastoma zhangbuensis, a New Species of the Subfamily Acrossocheilinae (Teleostei: Cyprinidae) from South China
Source: Animals (Basel). 2025 Aug 31;15(17):2563. doi: 10.3390/ani15172563 (PMC12427546; doi:10.3390/ani15172563)
Supplement: Supplementary file 1 [file animals-15-02563-s001.zip › animals-3761538-supplementary.pdf]

Supplemental materials for:

Hongiastoma zhangbuensis, a new species of the subfamily Acrossocheilinae (Teleostei: Cyprinidae) from south China

Table S1. Species list for phylogenetic analysis in this study.

Species	Sampling location	Nos.	References
**Acrossocheilinae**			
Acrossocheilus beijiangensis Wu & Lin 1977	Hechi, Guangxi Prov., China	KJ994641	[1]
Acrossocheilus fasciatus (Steindachner 1892)	N/A	NC023378	[2]
Acrossocheilus iridescens (Nichols & Pope 1927)	Baisha, Hainan Prov., China	KY244118	[3]
Acrossocheilus jishouensis Zhao, Chen & Li 1997	N/A	KY131974	N/A
Acrossocheilus kreyenbergii (Regan 1908)	Rongan, Guangxi Prov., China	KJ994646	[1]
Acrossocheilus kreyenbergii (Regan 1908)	Dongkou, Hunan Prov., China	KJ994650	[1]
Acrossocheilus longipinnis (Wu 1939) 1	Liuzhou, Guangxi Prov., China	KY244108	[3]
Acrossocheilus longipinnis (Wu 1939) 2	Rongan, Guangxi Prov., China	KJ994647	[1]
Acrossocheilus paradoxus (Günther 1868)	Yueyang, Hunan Prov., China	MF122060	[4]
Acrossocheilus parallens (Nichols 1931)	Libo, Guizhou Prov., China	KJ994628	[1]
Acrossocheilus spinifer Yuan, Wu & Zhang 2006	N/A	NC034918	N/A
Acrossocheilus wenchowensis Wang 1935	N/A	KC495074	N/A
Acrossocheilus wuyiensis Wu & Chen 1981	N/A	NC034919	N/A
Angustistoma barbatum (Lin 1931) 2	N/A	NC019630	N/A

Angustistoma barbatum (Lin 1931) 3	N/A	NC018043	[5]
Angustistoma barbatum (Lin 1931) 1	N/A	MH193215	N/A
Angustistoma macrolepis (Bleeker 1871)	Kangxian, Gansu Prov., China	MF122579	[4]
Folifer brevifilis (Peters 1881) 1	Hechi, Guangxi Prov., China	KJ994645	[1]
Folifer brevifilis (Peters 1881) 2	N/A	MH193229	N/A
Hongiastoma argentatum (Nguyen & Doan 1969) 1	Song Hong ecoregion, Vietnam	PQ665139	[6]
Hongiastoma argentatum (Nguyen & Doan 1969) 2	Song Hong ecoregion, Vietnam	PQ665140	[6]
Hongiastoma zhangbuensis sp. nov.1	Pingtang, Guizhou Prov., China	PV789635	This study
Hongiastoma zhangbuensis sp. nov. 2	Pingtang, Guizhou Prov., China	PV789636	This study
Hongiastoma zhangbuensis sp. nov. 3	Pingtang, Guizhou Prov., China	PV789637	This study
Onychostoma alticorpus (Oshima 1920) 1	Taiwan, China	KU942983	[7]
Onychostoma alticorpus (Oshima 1920) 2	Beinan River, Taiwan, China	NC021473	[8]
Onychostoma monticola (Günther 1888)	N/A	HM536893	[9]
Onychostoma ovale Pellegrin & Chevey 1936	Hechi, Guangxi Prov., China	KJ994643	[1]
Onychostoma rarum (Lin 1933)	N/A	KF626377	N/A
Onychostoma simum (Sauvage & Dabry de Thiersant 1874)	N/A	MZ870968	N/A
Onychostoma yunnanense (Regan 1904)	N/A	MZ870694	N/A
Scaphesthes barbatula (Pellegrin 1908)	Yongtai, Fujian Prov., China	KY653686	[10]
Scaphesthes brevibarba (Song, Cao & Zhang 2018)	Jiangyong, Hunan Prov., China	KY653704	[10]
Scaphesthes minnanensis (Jang-Liaw & Chen 2013)	N/A	MH193216	N/A
Scaphostoma gerlachi (Peters 1881)	Funing, Yunnan Prov., China	KJ994620	[1]
Scaphostoma krongnoense (Hoàng, Pham & Tran 2015)	N/A	MH193220	N/A
Scaphostoma lepturus (Boulenger 1900)	Wanquan River, Hainan Prov., China	NC054158	[11]
Scaphostoma meridionale (Kottelat 1998)	N/A	NC031603	N/A
**Barbinae**			
Barbus barbus (Linnaeus 1758)	N/A	ON097307	[12]

Barbus cyri De Filippi 1865	N/A	MW564419	N/A
Capoeta capoeta (Güldenstädt 1773)	N/A	HM536852	[9]
Capoeta razii Jouladeh-Roudbar, Eagderi, Ghanavi & Doadrio 2017	N/A	MF664707	[13]
Luciobarbus schejch Heckel 1843	Persian Gulf Basin, Iran	MF599077	[14]
Luciobarbus capito (Güldenstädt 1773)	N/A	MW564543	N/A
Luciobarbus esocinus Heckel 1843	Persian Gulf Basin, Iran	MF599074	[14]
Luciobarbus mursa (Güldenstädt 1773)	N/A	MW564480	N/A
Luciobarbus xanthopterus Heckel 1843	Persian Gulf Basin, Iran	MF599076	[14]
**Cyprininae**			
Cosmochilus harmandi Sauvage 1878	Champassak, Laos	JQ346163	[15]
Cyclocheilichthys armatus (Valenciennes 1842)	N/A	HM536926	[9]
Cyclocheilichthys janthochir (Bleeker 1854)	N/A	HM536907	[9]
Discherodontus ashmeadi (Fowler 1937)	N/A	JX066748	[16]
Discherodontus schroederi (Smith 1945)	N/A	JX066749	[16]
Scaphiodonichthys acanthopterus (Fowler 1934)	Menglun, Yunnan Prov., China	KJ994655	[1]
Scaphiodonichthys burmanicus Vinciguerra 1890	N/A	NC031605	N/A
Hypsibarbus malcolmi (Smith 1945)	N/A	HM536915	[9]
Hypsibarbus vernayi (Norman 1925)	Menglun, Yunnan Prov., China	KJ994615	[1]
Mystacoleucus lepturus Huang 1979	Puer, Yunnan Prov., China	KJ994654	[1]
Mystacoleucus obtusirostris (Valenciennes 1842)	Puer, Yunnan Prov., China	KJ994653	[1]
Poropuntius huangchuchieni (Tchang 1962)	Jiangcheng, Yunnan Prov., China	KJ994629	[1]
Poropuntius krempfi (Pellegrin & Chevey 1934)	Lvchun, Yunnan Prov., China	KJ994614	[1]
Poropuntius opisthopterus (Wu 1977)	Longling, Yunnan Prov., China	KJ994616	[1]

Puntioplites falcifer Smith 1929	N/A	HM536904	[9]
Puntioplites proctozysron (Bleeker 1864)	N/A	HM536912	[9]
Puntioplites waandersi (Bleeker 1859)	N/A	HM536928	[9]
Scaphognathops bandanensis Boonyaratpalin & Srirungroj 1971	N/A	HM536927	[9]
Scaphognathops stejnegeri (Smith 1931)	N/A	HM536906	[9]
Sikukia flavicaudata Chu & Chen 1987	Puer, Yunnan Prov., China	KJ994636	[1]
Sikukia gudgeri (Smith 1934)	Puer, Yunnan Prov., China	KJ994635	[1]
Sikukia stejnegeri Smith 1931	N/A	HM536898	[9]
**Labeoninae**			
Labeo dyocheilus (McClelland 1839)	N/A	JX074183	[16]
Labeo horie Heckel 1847	Alwero R, Ethiopia	JX074211	[16]
Labeo vulgaris Heckel 1847	Baro R., Gambella, Ethiopia	JX074220	[16]
**Schizothoracinae**			
Schizothorax oconnori Lloyd 1908	N/A	HM536902	[9]
Schizothorax lissolabiatus Tsao 1964	Baoshan, Yunnan Prov., China	KM611081	[17]
Schizothorax prenanti (Tchang 1930)	N/A	KY418130	N/A
Schizothorax progastus (McClelland 1839)	N/A	MK784999	N/A
Schizothorax richardsonii (Gray 1832)	Beas River, Himachal Pradesh, India	OR148188	[18]
Schizothorax waltoni Regan 1905	N/A	HM536903	[9]
**Spinibarbinae**			
Spinibarbus denticulatus Oshima 1926	Changjiang, Hainan Prov., China	KJ994631	[1]
Spinibarbus sinensis (Bleeker 1871)	N/A	HM536895	[9]
Spinibarbus yunnanensis (Tsü 1977)	Chengjiang, Yunnan Prov., China	KJ994652	[1]
**Torinae**			
Labeobarbus stenostoma (Boulenger 1910)	N/A	HM418183	N/A

Labeobarbus longidorsalis (Pellegrin 1935)	N/A	KT192925	N/A
Labeobarbus roylii (Boulenger 1912)	Lower Guinea, Congo	MK074391	[19]
Labeobarbus robertsi (Banister 1984)	Congo, Congo	MK074386	[19]
Labeobarbus progenys (Boulenger 1903)	Lower Guinea, Congo	MK074383	[19]
Labeobarbus macrolepidotus (Pellegrin 1928)	Congo, Congo	MK074381	[19]
Neolissochilus hexastichus (McClelland 1839)	Ranganadi River, Arunachal Pradesh, India	MK599508	[20]
Neolissochilus hendersoni (Herre 1940)	Penang Island, Malaysia	MW591117	[21]
Neolissochilus stracheyi (Day 1871)	Sangu, Bangladesh	MK572367	[22]
Tor malabaricus (Jerdon 1849)	Western Ghats, India	JX401295	N/A
Tor putitora (Hamilton 1822)	Ranganadi River, Arunachal Pradesh, India	MK599516	[20]
Tor tor (Hamilton 1822)	Northeast India	KT200174	N/A

Table S2. Genetic distance of COI gene among the species of *Angustistoma*, *Hongiastoma*, *Onychostoma*, *Scaphostoma*, and *Scaphesthes* within Acrossocheilinae.

		1	2	3	4	5	6	7	8	9	10	11	12
1	*Angustistoma barbatum*												
2	*Angustistoma barbatum*	0.0175											
3	*Angustistoma barbatum*	0.0175	0.0233										
4	*Angustistoma macrolepis*	0.0252	0.0214	0.0291									
5	*Onychostoma alticorpus*	0.1126	0.1068	0.1068	0.1126								
6	*Onychostoma alticorpus*	0.1126	0.1068	0.1068	0.1126	0.0000							
7	*Onychostoma monticola*	0.1087	0.1029	0.0951	0.1049	0.1049	0.1049						
8	*Onychostoma ovale*	0.1107	0.1010	0.0971	0.1068	0.0796	0.0796	0.0563					

9	*Onychostoma rarum*	0.1049	0.0951	0.0913	0.1010	0.0913	0.0913	0.0408	0.0466				
10	*Onychostoma simum*	0.1204	0.1068	0.1107	0.1165	0.0505	0.0505	0.0874	0.0796	0.0718			
11	*Onychostoma yunnanense*	0.1068	0.1010	0.0971	0.1029	0.0874	0.0874	0.0660	0.0641	0.0641	0.0699		
12	*Scaphesthes barbatula*	0.1029	0.0971	0.0971	0.0951	0.1243	0.1243	0.1146	0.0971	0.1107	0.1262	0.1146	
13	*Scaphesthes brevibarba*	0.1010	0.0913	0.0874	0.0932	0.0893	0.0893	0.1107	0.0971	0.1029	0.0913	0.0971	0.0893
14	*Scaphesthes minnanensis*	0.0893	0.0874	0.0796	0.0893	0.1049	0.1049	0.0971	0.0874	0.0932	0.1029	0.0854	0.0621
15	*Scaphostoma gerlachi*	0.1243	0.1223	0.1184	0.1204	0.1204	0.1204	0.1223	0.1107	0.1282	0.1184	0.1087	0.1165
16	*Scaphostoma krongnoense*	0.1146	0.1126	0.1087	0.1146	0.1204	0.1204	0.1165	0.1107	0.1243	0.1146	0.1126	0.0951
17	*Scaphostoma lepturus*	0.1204	0.1146	0.1029	0.1107	0.1204	0.1204	0.1107	0.1029	0.1126	0.1184	0.1087	0.0971
18	*Scaphostoma meridionale*	0.1126	0.1107	0.1029	0.1087	0.1223	0.1223	0.1126	0.1087	0.1184	0.1165	0.0990	0.1010
19	*Hongiastomata argentatum*	0.1437	0.1340	0.1417	0.1437	0.1165	0.1165	0.1301	0.1087	0.1262	0.1087	0.1029	0.1320
20	*Hongiastomata argentatum*	0.1437	0.1340	0.1417	0.1437	0.1165	0.1165	0.1301	0.1087	0.1262	0.1087	0.1029	0.1320
21	*Hongiastoma zhangbuensis*	0.1301	0.1165	0.1165	0.1262	0.1146	0.1146	0.1068	0.1010	0.1107	0.0990	0.0990	0.1262
22	*Hongiastoma zhangbuensis*	0.1320	0.1184	0.1184	0.1282	0.1165	0.1165	0.1087	0.1029	0.1126	0.1010	0.1010	0.1282
23	*Hongiastoma zhangbuensis*	0.1301	0.1165	0.1165	0.1262	0.1146	0.1146	0.1068	0.1010	0.1107	0.0990	0.0990	0.1262
		13	14	15	16	17	18	19	20	21	22	23	
1	*Angustistoma barbatum*												
2	*Angustistoma barbatum*												
3	*Angustistoma barbatum*												
4	*Angustistoma macrolepis*												
5	*Onychostoma alticorpus*												

6	*Onychostoma alticorpus*										
7	*Onychostoma monticola*										
8	*Onychostoma ovale*										
9	*Onychostoma rarum*										
10	*Onychostoma simum*										
11	*Onychostoma yunnanense*										
12	*Scaphesthes barbatula*										
13	*Scaphesthes brevibarba*										
14	*Scaphesthes minnanensis*	0.0583									
15	*Scaphostoma gerlachi*	0.1107	0.0971								
16	*Scaphostoma krongnoense*	0.1165	0.0951	0.0699							
17	*Scaphostoma lepturus*	0.1087	0.0874	0.0777	0.0796						
18	*Scaphostoma meridionale*	0.1107	0.0893	0.0641	0.0136	0.0816					
19	*Hongiastomata argentatum*	0.1204	0.1243	0.1184	0.1243	0.1146	0.1243				
20	*Hongiastomata argentatum*	0.1204	0.1243	0.1184	0.1243	0.1146	0.1243	0.0000			
21	*Hongiastoma zhangbuensis*	0.1087	0.1146	0.1301	0.1204	0.1320	0.1204	0.1049	0.1049		
22	*Hongiastoma zhangbuensis*	0.1107	0.1165	0.1320	0.1223	0.1340	0.1223	0.1068	0.1068	0.0019	
23	*Hongiastoma zhangbuensis*	0.1087	0.1146	0.1301	0.1204	0.1320	0.1204	0.1049	0.1049	0.0000	0.0019

References

1. Zheng, L.-P.; Yang, J.-X.; Chen, X.-Y. Molecular phylogeny and systematics of the Barbinae (Teleostei: Cyprinidae) in China inferred from mitochondrial DNA sequences. *Biochem. Syst. Ecol.* **2016**, *68*, 250–259. https://doi: 10.1016/j.bse.2016.07.012.

2. Cheng, S.H.; Yan, J.J.; Liu, Y.L.; Lu, Y.M.; Zhang, Y.; Xia, M.N.; Yan, Y.Z. The complete mitochondrial genome of Acrossocheilus fasciatus (Cyprinidae, Barbinae). *Mitochondrial DNA* **2015**, *26*, 941–942. PMID: 24409932.

3. Zheng, L.-P.; Yang, J.-X. Genetic diversity and population demography of the endemic species *Acrossocheilus longipinnis* (Teleostei, Cyprinidae) based on mtDNA COI and cyt b gene sequences. *Mitochondrial DNA A*. **2018**, *29*, 403–408. https://doi: 10.1080/24701394.2017.1292504. PMID: 28278689.

4. Shen, Y.; Hubert, N.; Huang, Y.; Wang, X.; Gan, X.; Peng, Z.; He, S. DNA barcoding the ichthyofauna of the Yangtze River: insights from the molecular inventory of a mega-diverse temperate fauna. *Mol. Ecol. Resour*. **2019**, *19*, 1278–1291. https://doi.org/10.1111/1755-0998.12961. PMID: 30375755.

5. Huang, Y.; Zhao, G.; Peng, Z. Mitochondrial genome of Onychostoma lini (Teleostei, Cypriniformes). *Mitochondrial DNA* **2012**, 23(3), 173–175.

6. Hoang, H.D.; Jang-Liaw, N.-H.; Pham, H.M.; Tran, N.T.; Durand, J.-D.; Nguyen, T.D.; Pfeiffer, J.; Page, L.M. Generic revision of the Southeast and East Asian torrent carp Subfamily Acrossocheilinae (Pisces: Teleostei) with description of three new genera and a new species from Vietnam. *J. Zool. Syst. Evol. Res*. **2025**, *2025*, 8895501. https://doi.org/10.1155/jzs/8895501.

7. Chang, C.-H.; Shao, K.-T.; Lin, H.-Y.; Chiu, Y.-C.; Lee, M.-Y.; Liu, S.-H.; Lin, P.-L. DNA barcodes of the native ray-finned fishes in Taiwan. *Mol. Ecol. Resour*. **2017**, *17*, 796–805. https://doi.org/10.1111/1755-0998.12601. PMID: 27717215.

8. Cheng, H.L.; Wu, C.Y.; Tsai, K.C.; Liu, A.Y.; Chiu, Y.W.; Tseng, D.Y. The complete mitochondrial genome sequence of Onychostoma alticorpus (Cypriniformes, Cyprinidae). *Mitochondrial DNA* **2014**, *25*, 188–189. https://doi.org/10.3109/19401736.2013.792069.

9. Yang, L.; Mayden, R.L.; Sado, T.; He, S.; Saitoh, K.; Miya, M. Molecular phylogeny of the fishes traditionally referred to Cyprinini sensu stricto (Teleostei: Cypriniformes). *Zool. Sci*. **2010**, *39*, 527–550.

10. Song, X.L.; Cao, L.; Zhang, E. Onychostoma brevibarba, a new cyprinine fish (Pisces: Teleostei) from the middle Chang Jiang basin in Hunan Province, South China. *Zootaxa* **2018**, *4410*, 147–163. https://doi.org/10.11646/zootaxa.4410.1.8. PMID: 29690161.

11. Zhai, D.; Xie, Z.; Wang, Y.; Yu, J.; Chen, Y.; Xia, M.; Liu, H.; Xiong, F. Complete mitochondrial genome of *Onychostoma leptura* and phylogenetic analysis of *Onychostoma. Mitochondrial DNA B Resour*. **2020**, *5*, 2297–2298.

12. Zangl, L.; Schaeffer, S.; Daill, D.; Friedrich, T.; Gessl, W.; Mladinic, M.; Sturmbauer, C.; Wanzenboeck, J.; Weiss, S.J.; Koblmueller, S. A comprehensive DNA barcode inventory of Austria's fish species. *PLoS One* **2022**, *17*, e0268694. https://doi.org/10.1371/journal.pone.0268694. PMID: 35679240.

13. Zareian, H.; Esmaeili, H.R.; Gholamhosseini, A.; Japoshvili, B.; Ozulug, M.; Mayden, R.L. Diversity, mitochondrial phylogeny, and ichthyogeography of the *Capoeta capoeta* complex (Teleostei: Cyprinidae). *Hydrobiologia* **2018**, *806*, 363–409.

14. Khaefi, R.; Esmaeili, H.R.; Amini Chermahini, M. Natural hybridization of Luciobarbus barbulus x Luciobarbus kersin and Luciobarbus barbulus x Luciobarbus xanthopterus in the Persian Gulf Basin. Turk. J. Fish. *Aquat. Sci*. **2018**, *18*, 1399–1407. https://doi.org/10.4194/1303-2712-v18_12_08.

15. Pasco-Viel, E.; Veran, M.; Viriot, L. Bleeker was right: Revision of the genus Cyclocheilichthys (Bleeker 1859) and resurrection of the genus Anematichthys (Bleeker 1859), based on morphological and molecular data of Southeast Asian Cyprininae (Teleostei, Cypriniformes). *Zootaxa* **2012**, *3586*, 41–54.

16. Yang, L.; Hirt, M. V.; Sado, T.; Arunachalam, M.; Manickam, R.; Tang, K.L.; Simons, A.M.; Wu, H.-H.; Mayden, R.L.; Miya, M. Phylogenetic placements of the barbin genera *Discherodontus*, *Chagunius*, and *Hypselobarbus* in the subfamily Cyprininae (Teleostei: Cypriniformes) and their relationships with other barbins. *Zootaxa* **2012**, *3586*, 26–40.

17. Chen, W.; Ma, X.; Shen, Y.; Mao, Y.; He, S. The fish diversity in the upper reaches of the Salween River, Nujiang River, revealed by DNA barcoding. *Sci Rep*. **2015**, *5*, 17437. https://doi: 10.1038/srep17437. PMID: 26616046.

18. Modeel, S.; Negi, R.K.; Sharma, M.; Dolkar, P.; Yadav, S.; Siwach, S.; Yadav, P.; Negi, T. A comprehensive DNA barcoding of Indian freshwater fishes of the Indus River system, Beas. *Sci. Rep*. **2024**, *14*, 2763. https://doi: 10.1038/s41598-024-52519-0. PMID: 38307873.

19. Sonet, G.; Snoeks, J.; Nagy, Z.T.; Vreven, E.; Boden, G.; Breman, F.C.; Decru, E.; Hanssens, M.; Ibala Zamba, A.; Jordaens, K.; Mamonekene, V.; Musschoot, T.; Van Houdt, J.; Van Steenberge, M.; Lunkayilaki Wamuini, S.; Verheyen, E. DNA barcoding fishes from the Congo and the Lower Guinean provinces: Assembling a reference library for poorly inventoried fauna. *Molec. Ecol. Resour*. **2019**, *19*, 728–743. https://doi:

10.1111/1755-0998.12983. PMID: 30576073.

20. Pandey, P.K.; Singh, Y.S.; Tripathy, P.S.; Kumar, R.; Abujam, S.K.; Parhi, J. DNA barcoding and phylogenetics of freshwater fish fauna of Ranganadi River, Arunachal Pradesh. *Gene* **2020**, *754*, 144860. https://doi: 10.1016/j.gene.2020.144860. PMID: 32531457.

21. Jamaluddin, J.A.F.; Lavoué, S.; Mohd Abu Hassan alshari, N.F.; Ghazali, S.Z.; Ahmad, A.; Tan, A.; Leng, C.L.; Mohd Nor, S.A. Reassessing fish diversity of Penang Island's freshwaters (northwest Peninsular Malaysia) through a molecular approach raises questions on its conservation status. *Biodiv. Conserv*. **2022**, *31*, 1551–1576.

22. Rahman, M. M.; Noren, M.; Mollah, A.R.; Kullander, S.O. Building a DNA barcode library for the freshwater fishes of Bangladesh. *Sci. Rep*. **2019**, *9*, 9382. https://doi: 10.1038/s41598-019-45379-6. PMID: 31253861.
